# Research Progress of Takeda G Protein-Coupled Receptor 5 in Metabolic Syndrome

**DOI:** 10.3390/molecules28155870

**Published:** 2023-08-04

**Authors:** Xianmei Gou, Lin Qin, Di Wu, Jian Xie, Yanliu Lu, Qianru Zhang, Yuqi He

**Affiliations:** 1 Guizhou Engineering Research Center of Industrial Key-Technology for Dendrobium Nobile, Zunyi Medical University, Zunyi 563000, China; 2 Joint International Research Laboratory of Ethnomedicine of Ministry of Education, Zunyi Medical University, Zunyi 563000, China

**Keywords:** bile acids, TGR5, Takeda G protein-coupled receptor 5, metabolic syndrome

## Abstract

Bile acids are acknowledged as signaling molecules involved in metabolic syndrome. The Takeda G protein-coupled receptor 5 (TGR5) functions as a significant bile acid receptor. The accumulated evidence suggests that TGR5 involves lipid homeostasis, glucose metabolism, and inflammation regulation. In line with this, recent preclinical studies also demonstrate that TGR5 plays a significant role in the generation and progression of metabolic syndrome, encompassing type 2 diabetes mellitus, obesity, atherosclerosis, and non-alcoholic fatty liver disease (NAFLD). In this review, we discuss the role of TGR5 in metabolic syndrome, illustrating the underlying mechanisms and therapeutic targets.

## 1. Introduction

Metabolic syndrome (MS) refers to a group of metabolic dysregulations characterized by central obesity, hyperglycemia, insulin resistance, and hypertension. Individuals with MS have an increased risk of cardiovascular diseases and type 2 diabetes mellitus (T2DM) [1,2]. MS has become an increasing global public health problem, with the worldwide prevalence of metabolic syndrome predicted to encompass one-fourth of the world’s population, equating to an excess of one billion people worldwide being affected by metabolic syndrome [3,4]. It is most significant for lowering blood glucose and lipid levels of MS individuals in the therapeutic and preventive aspects of cardiovascular diseases.

**Figure 1:**
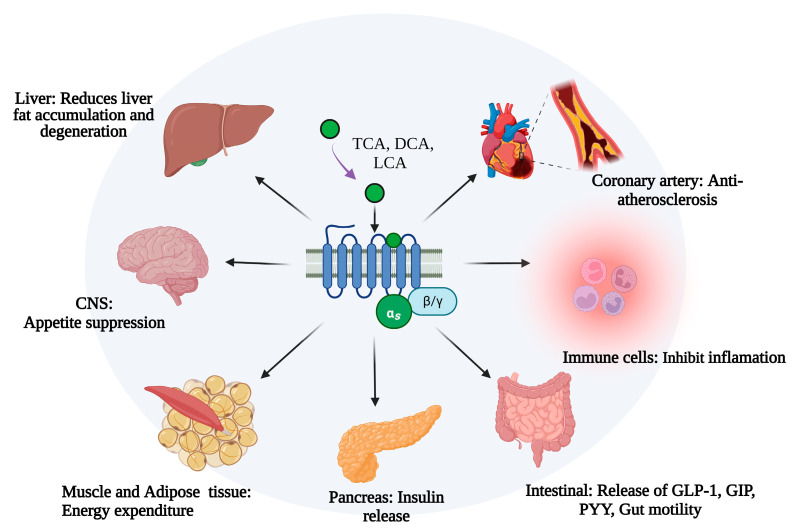
The role of TGR5 in metabolic syndrome in different cells and tissues. TCA, taurocholic acid; DCA, deoxycholic acid; LCA, Lithocholic acid; CNS, center nervous system; NAFLD, non-alcoholic fatty liver disease; NASH, non-alcoholic steatohepatitis; GLP-1, glucagon-like peptide-1; GIP, gastric inhibitory polypeptide; PYY, Peptide YY. This figure was created by Biorender (https://app.biorender.com/, accessed on 9 June 2023).

Takeda G protein-coupled receptor 5 (TGR5, GPBAR1, M-BAR, or GPR131) is a bile acid membrane receptor and a member of the G protein-coupled receptor subfamily. TGR5 can influence bile acid composition and enterohepatic circulation [5]. Accumulated evidence has demonstrated that TGR5 plays special roles in lipid and glucose metabolism, energy homeostasis, and immune and anti-inflammatory regulation [6,7,8,9]. TGR5 is widely expressed in the liver, intestine, pancreas, central nervous system, and immune cells [10]. TGR5 activation by bile acids can influence lipid, glucose, and energy metabolism (Figure 1). Hepatic TGR5 acts as a suppressor of lipid accumulation in the liver by regulating the bile acids composition and enterohepatic circulation [11,12]. Mouse models with MS have illustrated that the activation of TGR5 could promote insulin secretion and insulin sensitivity [13,14]. TGR5 is also involved in neuronal regulation in the central system to influence food intake and body weight [15]. Studies using tissue-specific mice have revealed that TGR5 could increase energy expenditure in skeletal muscle and adipose tissue [16,17]. TGR5 also involves in the pathogenesis of atherosclerosis by regulating the secretion of vasodilators and vasoconstrictors [18,19] (Figure 1).

Bile acids (BAs) have been acknowledged as pivotal signal factors involving diverse biological functions including metabolic homeostasis and the immune response, mainly through activating two bile acid receptors: TGR5 and farnesoid X receptor (FXR). Recently, hundreds of research articles about TGR5 and MS have been collected in SCIE and PubMed up to 2023. However, the approved drugs targeting TGR5 for the treatment of MS are still limited. This review aims to map the underlying mechanisms of TGR5 and its potential as a drug target in the treatment of MS.

## 2. TGR5

The encoded sequence of the TGR5 gene is evolutionarily conserved in humans, rats, and other mammals and is located on human chromosome 2q35 and mouse chromosome 1c3 [6,20]. TGR5 gene expression is densely found in human tissues and cells, including the brain, liver, endocrine glands, skeletal muscles, spleen, lymph nodes, lung, placenta, nerve cells, and adipocytes. In the liver, TGR5 is highly expressed in bile duct cells, activated liver stem cells, sinusoidal endothelial cells, Kupffer cells, and NKT cells [10,21].

There are seven transmembrane domains (TMH1-7) of TGR5, including three extracellular loops that bind to ligands (ECL1-3) and three intracellular loops responsible for transmitting signals (ICL1-3) [22]. TGR5 is activated by bile acids in the order of LCA > DCA > CDCA > CA [23]. Bile acids and TRG5 agonists interact with TGR5 in the binding pocket and deliver a G-protein complex with α_s_, β, and γ subunits [24]. GDP is subsequently released from the G protein complex and replaced by GTP, inducing the separation of G-protein α_s_ with β-γ dimers. G-protein α_s_ activates intracellular adenylyl cyclase (AC), resulting in ATP to cAMP conversion and protein kinase A (PKA) activation, which further induce downstream signaling involving bile acid synthesis, insulin sensitivity, atherosclerosis development, energy expenditure, and inflammation [25,26].

## 3. TGR5 and Bile Acids

**Figure 2:**
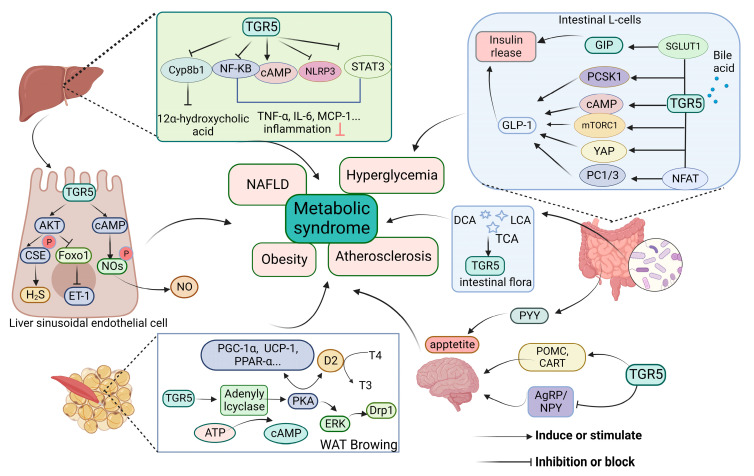
Review on the mechanisms of TGR5 in metabolic syndrome. TGR5 signaling is involved in the regulation of bile acid synthesis, vascular factor function [11,18], amelioration of inflammatory diseases by inhibiting inflammation [30,35,36], modulation of endocrine peptide release to ameliorate glucose disorders [17,37,38], appetite suppression, and promotion of energy expenditure [39,40]. TGR5 is also indirectly activated by regulating intestinal flora [41,42]. TGR5, takeda G protein-coupled receptor 5; TCA, taurocholic acid; DCA, deoxycholic acid; LCA, lithocholic acid; CYP8B1, cholesterol 12α hydroxylase; cAMP, cyclic adenosine monophosphate; PKA, protein kinase A; GLP-1, glucagon-like peptide-1; PC1/3, prohormone convertase 1/3; PCSK1, proprotein convertase subtilisin/kexin type 1; mTOR, mammalian target of rapamycin; NFAT, nuclear factor of activated T cells; YAP, yes associated protein; GIP, gastric inhibitory polypeptide; SGLT1, sodium glucose cotransporter 1; PYY, peptide YY; TNF-α, tumor necrosis factor-α; IL-6, interleukin-6; NLRP3, NOD-like receptor domain associated protein 3; WAT, white adipose tissue; ERK, extracellular regulated protein kinases; Drp1, dynamin-related protein 1; D2, deiodinase 2; T4, thyroxine; T3, 3,5,3′-triiodothyronine; Ucp1, uncoupling protein 1; PGC-1α, PPAR-γ coactivator-1α; POMC, pro-opiomelanocortin; CART, cocaine-amphetamine-regulated transcript; YAgRP/NPY, neuropeptide Y/agouti-related peptide; MCP-1, monocyte chemoattractant protein-1; NOS, nitric oxide synthase; NO, nitric oxide; STAT3, signal transducer and activator of transcription 3; CSE, cystathionine γ-lyase; FoxO1, forkhead box protein O1; ET-1, endothelin-1. This figure was created by Biorender (https://app.biorender.com/, accessed on 11 June 2023).

BAs, including primary and secondary types, are synthesized from cholesterol and other precursors within hepatocytes. BAs have been acknowledged as metabolic messengers that could regulate their own metabolism and immune system by targeting FXR and TGR5 [27,28]. A disruption of the bile acids pool component has been described in MS, including T2DM, insulin-resistant, and non-alcoholic hepatitis [29,30,31]. Cholesterol 12α hydroxylase (CYP8B1) is an important enzyme in the synthesis of bile acids, particularly with the amount of 12α-hydroxy bile acids. TGR5 activation decreases the expression of CYP8B1 and reduces the ratio of 12α-hydroxylated bile acids to non-12α-hydroxylated bile acids in the bile acid pool. Mcgavgan et al. [11] found that reduced circulating bile acid profile hydrophobicity was related to a TGR5-dependent expression of the CYP8B1 protein. Meanwhile, the TGR5 agonist (INT-777) could downregulate hepatic Cyp8b1 expression [32]. The levels of Cyp8b1 and hydrophobic bile acids, especially 12α-hydroxy bile acids, have been upregulated in tgr5^−/−^ mice and significantly reduced in tgr5^+/+^ mice [33,34]. Thus, selective under-regulation of the expression level of hepatic Cyp8b1 may be a mechanism by which TGR5 regulates the bile acid profiles (Figure 2).

## 4. TGR5 and T2DM

TGR5 is an essential receptor in maintaining the balance of glucose levels in the body and controlling insulin sensitivity. It was suggested that TGR5^−/−^ mice display impaired glucose tolerance and insulin resistance compared to their WT counterparts, whereas TGR5-overexpressing mice showed good tolerance to glucose [9]. Meanwhile, the administration of TGR5 agonists was shown to modulate glucose levels in high-fat diet (HFD) mice, further demonstrating that TGR5 plays an active role in the disease process of T2DM [43].

### 4.1. Promote GLP-1 Secretion

Several studies suggest that TGR5 activation elevates glucagon-like peptide-1 (GLP-1) secretory capacity and expands the L-cell population (Figure 2). The intestinal L cells are the main synthesizing and releasing cells of GLP-1, and TGR5 sits in the basal layer of L cells [44]. GLP-1 is an important peptide hormone that binds to the GLP-1 receptor in pancreatic β cells; its functions include stimulating insulin release, delaying gastric emptying, enhancing satiety, and inhibiting glucagon secretion [45].

On the one hand, TGR5 was found to induce GLP-1 production and regulate glucose metabolism through different metabolic pathways. In STC-1 cells, handling of bile acid-activated TGR5 stimulated GLP-1 secretion through the cAMP pathway [13]. Prohormone convertase 1/3 (PC1/3), processing and modifying GLP-1, is an important enzyme for the production and secretion of GLP-1. It has been found that altered intestinal bile acid composition induced an elevation of the TGR5 agonist, taurocholic acid (TCA), which stimulated the nuclear factor of activated T cells (NFAT). Then, PC1/3 binds to NFAT binding, promotes PC1/3 gene expression, and induces GLP-1 secretion, thereby improving glucose tolerance [46]. Meanwhile, proprotein convertase subtilisin/kexin type 1 (PCSK1) encoding the PC1/3 is important for GLP-1 synthesis and can affect its secretion. It was shown that the administration of oligofructose (OFS) to wild-type mice improved glucose tolerance compared to TGR5^−/−^ mice. The mechanism was as follows: OFS altered the composition of the intestinal BAs, particularly 6α-hydroxy bile acids, increased TGR5 activity, and promoted PCSK1 expression and, subsequently, GLP-1 release [47]. mTORC1 is a complex of mammalian target of rapamycin (mTOR). Several studies have shown that ileal TGR5–mTOR1 signaling engages in GLP-1 secretion in L cells. The cellular experiment showed that deoxycholic acid (DCA) activated the TGR5–mTOR1 signaling pathway, phosphorylated mTOR, S6, and S6 kinases (S6Ks), and induced GLP-1 synthesis and release. However, the effect of DCA-induced GLP-1 synthesis is abolished when siRNA is knocked down of mTORC1 or TGR5 [48].

On the other hand, TGR5 could expand the L-cell population indirectly to increase GLP-1 production. Ginsenoside K has been found to activate TGR5 by elevating lithocholic acid (LCA) and DCA content. This, in turn, activates the RhoA/ROCK/YAP signaling pathway, increasing the abundance of L-cells, and ultimately enhancing GLP-1 release [49]. When TGR5 is knocked down, yes-associated protein (YAP) exhibits low expression and loses its glucose regulatory function [50]. Therefore, TGR5 is a promising target for use against T2DM.

FXR and TGR5 are essential receptors in the feedback of bile acids synthesis and en-terohepatic circulation [51]. Moreover, these two receptors are co-expressed in the ileum and colon [52]. In intestinal endocrine L cells, FXR and TGR5 could stimulate GLP-1 secretion, which contributes to increasing insulin secretion to improve insulin sensitivity, respectively. A study using FXR/TGR5-knockout mice has illustrated that FXR and TGR5 may coordinately regulate GLP-1 production and secretion [53]. Further research on the roles of FXR and TGR5 in metabolic syndrome is still needed.

### 4.2. Promote GIP Secretion

A glucose-dependent gastric inhibitory polypeptide (GIP) increases insulin secretion and maintains glucose homeostasis in the intestine (Figure 2). Upon food ingestion, intestinal cells release GIP into the bloodstream, where it binds to the GIP receptor (GIPR) on pancreatic β-cells. This activation of GIPR leads to increased cAMP levels and proinsulin gene transcription, which ultimately promotes insulin secretion and regulates the survival and reproduction of insulin-independent pancreatic β cells [37]. Zhu et al. [54] found that saponins derived from *Camellia Sinensis* seeds could stimulate GIP secretion in mouse and STC-1 cells via sodium glucose cotransporter 1 (SGLT1) and TGR5. However, TGR5 siRNA was able to counteract the increase in GIP expression, revealing that TGR5 participates in the regulation of GIP expression for the first time. Furthermore, network pharmacology analysis showed that PCSK1 was related to SGLT1, TGR5, and GIP [54]. Interestingly, the downregulation of PCSK1 inhibits GIP and GLP-1 production in STC-1 cells [55]. Thus, the TGR5–PCSK1 axis is likely critical for the simultaneous induction of GLP-1 and GIP secretion, and in vivo experiments are still needed to verify the involved mechanisms further. The TGR5–GIP signaling pathway offers a promising avenue for creating novel hypoglycemic medications.

### 4.3. Promote PYY Secretion

Peptide YY (PYY) is a gastrointestinal hormone released by L cells in the ileum and colon. PYY could slow gastric emptying, delay gastrointestinal transport, and suppress appetite through the hypothalamic circuits [56]. Bile acids promote the secretion of PYY by activating the TGR5 receptor in L cells (Figure 2). In vitro studies have shown that the TGR5 agonist DCA induces the release of PYY in a dose-dependent manner [23]. However, this effect was not observed in TGR5 knockout mice [57]. In a clinical study, the administration of DCA and TCA can simulate a rapid and noticeable increase in plasma PYY. In contrast, applying bile acids with a low binding affinity to TGR5 cannot induce PYY secretion significantly [58]. Of mechanistic relevance was the demonstration that activation of TGR5 stimulates the production of Gα and cAMP to release PYY via the Epac/PLC-ε-CA^2+^ pathway in a cAMP-dependent manner [59]. The findings above demonstrate that TGR5 signaling is associated with bile acid-induced PYY secretion.

### 4.4. Increase Glucose Metabolism in Skeletal Muscle

TGR5 is highly expressed in skeletal muscle, the largest organ of glucose handling in humans. Skeletal muscle also increases glucose clearance and improves insulin resistance to regulate body glucose homeostasis, in addition to promoting energy metabolism (Figure 2). One TGR5 agonist, known as 4-Phenoxynicotinamide (MN6), could enhance insulin-stimulated glucose transport via the cAMP/PKA pathway, thereby ameliorating insulin resistance and glucose tolerance in skeletal muscle in HFD mice and C2C12 myotubes [60]. Furthermore, TGR5 has been proven to alleviate glucose homeostasis and age-related glucose disorders in skeletal muscle. Sasaki et al. [17] found that the activation of the glycolytic pathway in skeletal muscle-specific TGR5 transgenic mice led to increased glucose utilization and clearance in HFD mice. Moreover, observation shows that old transgenic mice exhibit increased the glucose clearance and strength of the skeletal muscles. More importantly, the activation of TGR5 increases a higher muscle mass, which is beneficial in improving diabetes. Sasaki et al. [21] showed that exercise-induced unfolded protein response (UPR) and activating transcription factor 6α (ATF6α) increase the expression of TGR5, which, in turn, promotes the differentiation of skeletal muscle cells and muscle hypertrophy in mice. Moreover, Sund et al. [61] found that TGR5 strengthens skeletal muscle regeneration and myogenic process through the AKT/mTOR/FoXO3 signaling pathway in C2C12 cells. This research reveals that activating TGR5 in skeletal muscle effectively improves glucose metabolism disorder, opening new avenues for drug development to treat diabetes.

### 4.5. Inhibit Inflammation

Chronic inflammation is a crucial initiating factor of insulin resistance. TGR5 signaling becomes a promising approach for treating T2DM by interfering with the inflammatory response (Figure 2). Several studies have shown a high expression of TGR5 in monocytes, macrophages, and immune organs such as the spleen. The bile acid–TGR5 pathway inhibits the secretion of various cytokines, including tumor necrosis factor-α (TNF-α) and interleukin (IL)-1α, IL-1β, IL-6, and IL-8 [62].

In Kupffer cells, TGR5 activation reduces cytokine expression induced by LPS and inhibits NF-κB-dependent inflammatory responses, improving glucose tolerance [63]. According to a study, activation of TGR5 reduces chemokines expression in macrophages via the AKT–mTOR pathway [64]. This pathway involves the phosphorylation of AKT and mTORC1, which induces the expression of 4E-binding proteins (4E-BP) and CCAAT/enhancer binding protein beta (C/EBPβ) isoform liver-inhibitory protein (LIP). Therefore, the TGR5–AKT–mTOR-LIP pathway is a newly discovered pathway for macrophages to promote anti-diabetic effects [64]. Moreover, abnormal glucose metabolism is closely associated with regulating NOD-like receptor domain-associated protein 3 (NLRP3) inflammatory vesicles. TGR5 activation induces PKA kinase, leading to the ubiquitination of NLRP3. Therefore, the TGR5–cAMP–PKA axis inhibits the formation of NLRP3 inflammatory vesicles to attenuate inflammatory response, glucose tolerance, and insulin resistance induced by a high-fat diet [65]. It also estimated that the inflammation and insulin resistance of chimeric mice simultaneous upregulated after that specifically removing TGR5 from macrophages and bone marrow [26]. According to these studies, TGR5 signaling could prevent hepatic insulin resistance development and progression, ultimately improving systemic glucose levels.

## 5. TGR5 and Obesity

### 5.1. Promote Energy Consumption and Fat Browning

Obesity is mainly induced by an imbalance between energy intake and expenditure, ultimately resulting in increased fat storage in adipose tissue. Adipose tissue can be divided into white adipose tissue (WAT) and brown adipose tissue (BAT). WAT primarily stores energy, while BAT contains more mitochondria and plays a key role in energy consumption. Therefore, an increase in BAT is conducive to improving obesity. TGR5 is a crucial target for regulating mitochondrial function and promoting adipose tissue remodeling (Figure 2).

BAs haven been acknowledged as metabolic regulators affecting energy expenditure via TGR5 stimulation. In brown adipose tissue and skeletal muscle, the interaction of BA and TGR5 promotes the activation of type 2 deiodinase (Dio2), which induces the expression of the crucial metabolic genes including uncoupling protein 1 (UCP1) and peroxisome proliferator-activated receptor-gamma coactivator-1 alpha (PGC-1α) in mitochondria [66,67,68]. Dio2 converts inactive thyroxine (T4) to active 3,5,3′-triiodothyronine (T3), inducing the activation of UCP1 [16,69]. Mitochondrial UCP1 diverts the electron gradient through proton leak and induces uncoupled respiration [70,71]. PGC–1α co-activates proliferator-activated receptor γ (PPARγ) and fine-tunes mitochondrial biogenesis, oxidative metabolism, and thermogenesis [72]. The thermogenic activity of BA supplementation was disappeared in Tgr5-knockout mice that were fed a high-fat diet [73]. BA–TGR5–Dio2 signaling promotes thermogenic activity instead of ATP production. In addition, an in-vivo study demonstrated administration of TGR5-activated bile acid, which can transform white adipose tissue into beige adipose tissue and promote mitochondrial fission via the extracellular regulated protein kinases/dynamin-related protein 1 (ERK/DRP1) pathway [70].

### 5.2. Increase Intestinal Peristalsis

TGR5 is involved in bile acids-induced intestinal peristalsis and significantly prevents central obesity caused by constipation. Studies have demonstrated that TGR5 agonists can enhance the intestinal motility of mice, while the absence of TGR5 results in a longer colonic transit time and decreased frequency of defecation in mice [50]. Activated TGR5 could stimulate the secretions of peristaltic mediators, including 5-hydroxytryptamine (5-HT) and calcitonin gene-related peptide (CGRP) in enterochromaffin cells (ECs), while the effect was lost in TGR5^−/−^ mice [74]. In vitro and in vivo experiments have demonstrated that TGR5 activation can enhance the production of 5-HT from EC cells. This improvement in constipation is achieved by activating the transient receptor potential ankyrin 1 (TRPA1) and the PLC-γ1/PIP2 signaling pathway [75].

### 5.3. Regulate Hypothalamic Neurons

TGR5 signaling in the hypothalamus is an important factor in protecting against obesity caused by HFD. Hypothalamic bile acid level is reduced in diet-induced obese (DIO) mice compared to the control group [15]. However, when DIO mice are administered with bile acids or central TGR5 agonists, their body weight and fat mass are reduced by activating the sympathetic nervous system, thereby regulating energy homeostasis. Conversely, when hypothalamic TGR5 expression in the medio basal hypothalamus is downregulated or deleted, it contributes to obesity by weakening sympathetic nerve activity [15]. Together, hypothalamic TGR5 is an important receptor for anti-obesity via the supplementation of bile acid, which provides a basis for the development of BA–TGR5 as a pathway for anti-obesity drugs.

Findings from a study of the central neurons involved in TGR5 regulation of food intake to improve obesity (Figure 2). TGR5 not only promotes the expression of anorexia neurons to increase satiety signals but also inhibits the expression of hunger-signaling neurons to suppress hunger signals, thereby reversing obesity. A study has shown that DCA and cholecystokinin octapeptide (CCK8) act synergistically to stimulate pro-opiomelanocortin (POMC) and cocaine–amphetamine-regulated transcript (CART) neurons via the TGR5 receptor of the valgus nerve. Then, it delivers satiety signals to the hypothalamus, reducing food intake [76]. Chen et al. [77] investigated the mechanism of bile acids releasing POMC in mouse hypothalamus GT1-7 cells. The results showed that phosphorylation of the signal transducer and activator of transcription 3 (p-STAT3), phosphorylation of threonine kinase (p-AKT), suppressor of cytokine signaling (SOCS3), and TGR5 expression were significantly increased. This led to the conclusion that bile acid may regulate anorexigenic neuropeptides through p-AKT and p-STAT3–SOCS3 signaling. In the other study, TGR5 activation inhibits the secretion of hypothalamic appetite-promoting neuropeptide Y/agouti-related peptide (AgRP/NPY) neurons via the Rho/ROCK/Actin-remodeling pathway, which, in turn, suppresses hunger signaling to reduce food intake. In contrast, the deletion of TGR5 results in a notable rise in food consumption and encourages the onset of obesity [78]. In addition to POMC, CART, and AgRP/NPY neurons, other neurons still play a role in regulating body weight through TGR5 and related mechanisms. Further exploration is needed to understand their involvement fully.

## 6. TGR5 and Atherosclerosis

### 6.1. Regulate Inflammation

The development and progression of atherosclerosis are promoted by dyslipidemia and chronic inflammation, with TGR5 being associated with the pathogenesis of atherosclerosis. When macrophages remove the modified forms of LDL-C from the vascular wall, it eventually forms foam cells and produces local inflammatory factors and chemokines, causing chronic inflammation in the vascular wall and promoting lipid accumulation in the cells [24]. It has been shown that activation of TGR5 blunts the production of a cluster of differentiation 36 (CD36), scavenger receptor A (SR-A), monocyte chemoattractant protein-1 (MCP-1), and chemokine ligand-5 (CCL5) through the cAMP–NF-κB signaling pathway [79], which reduces the uptake of oxidized LDL (Ox-LDL) and the formation of foam cells, thereby inhibiting the progression of atherosclerosis. In the atherosclerosis model of LDL^−/−^ mice, activation of TGR5 reduces atheromatous plaques by inhibiting inflammation and lipid accumulation. However, when the Tgr5 gene was knocked out, there was no inhibitory effect on atherosclerosis [79]. This suggests that the activation of TGR5 plays a crucial role in preventing atherosclerosis by modulating the immune system.

### 6.2. Promote NO Release

Nitric oxide (NO) is an essential molecule in preventing atherosclerosis. It has multiple beneficial effects, including vasodilation and increased blood flow. Moreover, NO reduces the adhesion of monocytes to vascular endothelial cells, thus preventing the formation of atheromatous plaque. Activation of TGR5 may offer a novel therapeutic strategy for treating atherosclerosis. When bile acid binds to TGR5 and triggers the expression of cAMP in liver sinusoidal endothelial cells, this process results in an elevation of intracellular Ca^2+^ levels, leading to the activation of nitric oxide synthase (NOS) phosphorylation and subsequent release of nitric oxide (NO) (Figure 2) [80].

TGR5 is activated by endogenous bile acids, with TCA being the most effective, followed by LCA, DCA, chenodeoxycholic acid (CDCA), and cholic acids (CA). A study revealed that individuals with atherosclerosis had significantly lower levels of LCA in their serum. This reduction level of LCA could impede TGR5 on anti-inflammatory in macrophages, leading to increasing plaque formation. Thus, LCA may be a marker for the clinic diagnosis of coronary atherosclerosis [19].

## 7. TGR5 and NAFLD

Prior research has demonstrated that TGR5 protects against several liver diseases. Non-alcoholic fatty liver disease (NAFLD) is a common complication of obesity and T2DM. NAFLD has the potential to advance to non-alcoholic steatohepatitis (NASH), liver fibrosis, hepatocellular injury, cirrhosis, and hepatocellular carcinoma, because of lipid buildup in the liver (Figure 2). The TGR5 receptor may play a role in the regulation of NAFLD/NASH, as indicated by a notable decrease in TGR5 expression observed in the liver tissues of both NASH patients and NASH animals [35,65]. Meanwhile, TGR5^−/−^ HFD mice showed lipid accumulation and increased hepatic fat energy [11]. Furthermore, the impact of TGR5 on lipid accumulation may vary depending on the gender of the individual. Research conducted by Vassileva et al. [9] on DIO mice revealed that male Tgr5^−/−^ mice showed higher levels of hepatic steatosis than female Tgr5^−/−^ mice, highlighting the regulatory role of TGR5 in this process.

### 7.1. Improve Insulin Resistance

The development of NAFLD is significantly influenced by insulin resistance in both the liver and adipose tissue [81]. The TGR5 signaling pathway is critical in regulating intestinal GLP-1 secretion and improving insulin resistance. According to Lambert et al. [82], GLP-1 has been shown to improve insulin sensitivity and reduce hepatic glucose production in the animal model of NAFLD, which inhibits the development of liver steatosis, NASH, and liver fibrosis. A clinical study has further demonstrated that treatment with liraglutide, a GLP-1 agonist, can hinder the histological deterioration of NAFLD. This is achieved by reducing body weight and fasting blood glucose, increasing insulin sensitivity and inhibiting de novo lipogenesis and inflammation [83]. In addition, activation of TGR5 releases Glucagon-like peptide-2 (GLP-2), which possesses anti-inflammatory properties and accelerates liver regeneration after partial hepatectomy [84,85]. Thus TGR5 is expected to be a novel target that improves NAFLD/NASH via increasing the release of GLP-1 as well as GLP-2.

### 7.2. Inhibit Inflammation

Monocytes and macrophages are important sources of inflammatory factors in the liver, playing a crucial role in the development of NASH and hepatocellular carcinoma [86]. The TGR5 receptor improves NASH in various immune cells by inhibiting inflammation (Figure 2).

Activation of TGR5 in macrophages has been shown to effectively inhibit inflammatory responses through the toll-like receptor 4 (TLR4)/NF-κB pathway [87]. Additionally, TGR5 activation reduces the binding activity of NF–κB–p65 DNA, inhibiting the NF–κB pathway and decreasing TNF-α secretion in CD14 macrophages both in vitro and in vivo [88]. Moreover, TGR5 agonists increase the phosphorylation of PKA and induce ubiquitination of NLRP3 inflammasome, which inhibits NLRP3-mediated M1 macrophage polarization, thereby alleviating liver steatosis and inflammation in NASH [89]. Activation of TGR5 has been observed to inhibit lipopolysaccharide (LPS)-induced inflammatory factors production in Kupffer cells and THP-1 cells of TGR5 overexpressed. Additionally, TGR5 activation has been found to reduce phosphorylated level of the signal transducer and activator of transcription 3 (STAT3), resulting in a hepatoprotective effect [90]. Conversely, LPS-induced TGR5^−/−^ mice show severe liver injury, hepatocyte apoptosis, and inflammation and cause elevated serum alanine and aspartate aminotransferase [91]. The above studies show that TGR5 signaling pathways inhibit the occurrence and development of NASH by regulating inflammatory factors, providing a new idea for the treatment of NASH.

### 7.3. Ameliorate Effect on Fibrin

TGR5 agonists ameliorate liver fibrosis by inducing apoptosis, inhibiting inflammation, and improving blood circulation.

LCA has been identified as the effective natural agonist of TGR5, which plays a crucial role in regulating metabolism and inhibiting inflammation. A recent study has investigated the effects of LCA on immune cells and hematopoietic stem cells (HSCs) in liver fibrosis. According to the findings, LCA could hinder the activity of transforming growth factor beta (TGF-β) and glycolysis while enhancing oxidative phosphorylation. This leads to the differentiation of macrophages towards the M2 type and prevents their differentiation into the M1 type [92]. Meanwhile, LCA increases the recruitment of NK cells and decreases NKT cell activation, ameliorating liver fibrosis in mice induced by carbon tetrachloride (CCL4) [92]. BAR501, a TGR5 agonist, has been shown to reverse liver and vascular damage in mouse models of NASH in a TGR5-dependent manner. BAR501 treatment induces the browning of white adipose tissue and inhibits the development of aortic intima–media thickness [93]. Additionally, it reduces the expression of specific inflammatory genes and adhesion molecules in the aorta, such as IL-6, TNF-a, vascular cell adhesion molecule (VCAM), intercellular adhesion molecule-1 (ICAM-1), and endothelial selection [93].

The activation of TGR5 in liver sinusoidal endothelial cells (LSECs) promotes vasodilators secretion and inhibition of vasoconstrictors release (Figure 2). The former is achieved through the induction of endothelial nitric oxide synthase (eNOS) phosphorylation, which increases nitric oxide (NO) production [18]. Similarly, TGR5 activation increases cystathionine γ-lyase (CSE) activity via Akt-induced serine phosphorylation, increasing H_2_S production. Moreover, the activation of Akt leads to the reduced transcription of forkhead box protein O1 (FoxO1), inhibiting the expression of vasoconstrictor endothelin-1 (ET-1) [94]. These findings indicate that increased TGR5 signaling has the potential to improve liver function by decreasing lipid buildup in the liver, reducing fibrosis and inflammation.

## 8. Gut Microbiota Regulates TGR5

The interaction between gut flora and the TGR5 receptor has become a current research hotspot. Accumulating evidence shows that gut flora indirectly activates the TGR5 pathway by metabolizing the composition of the bile acid profile, facilitating the effect on a series of MS. In contrast, TGR5 deficiency causes gut microbiota imbalance and aggravates liver injury.

Studies have demonstrated that knocking out hypoxia-inducible factor 2α (HIF-2α) in mice can effectively reduce obesity by decreasing gut lactate levels (Figure 2). This reduction leads to a decrease in the frequency of *Bacteroides vulgatus* and an increase in the abundance of *Ruminococcus torques*. These changes result in higher TCA and DCA and TGR5 activation levels. Activation of TGR5 enhances the thermogenic capacity of white adipose tissue by increasing the expression of UCP-1 and creatine kinase mitochondrial 2 (CKMT2) [44]. A recent study has shown that flaxseed can alleviate NASH by reducing the abundance of *Firmicutes* and increasing the abundance of *Bifidobacterium* and *Actinobacteria*. Additionally, it can increase the proportion of LCA and DCA, which can inhibit liver inflammation and protect against HFD-induced hepatic steatosis via the TGR5–NF–κB signaling pathway [95]. Dietary supplementation with probiotics can enhance insulin sensitivity and stimulate adipose tissue browning, ultimately resulting in weight loss. The mechanism increases the abundance of *Bacteroides* that further induce the TGR5–GLP-1 pathway and inhibit intestinal dipeptidyl peptidase-4 (DDP4) [96]. Meanwhile, a clinical study has also shown that probiotics consumption improves metabolic parameters such as lowering cholesterol, improving intestinal function and increasing insulin sensitivity in obese and T2DM patients [41].

## 9. TGR5 Agonists

TGR5 activation could improve insulin resistance, increase adipose tissue thermo-genesis and energy expenditure, and inhibit inflammation. However, the approved drug targeting TGR5 is limited in the treatment of MS. Bile acid analogs and non-bile acid regulators exhibit therapeutic potential for the treatment of metabolic disorders, including obesity, diabetes, and NAFLD. Ursodeoxycholic acid (UDCA) is a secondary bile acid used as a first-line drug in the treatment of primary biliary cholangitis [97]. A clinical study has shown that administering low doses of UDCA can enhance GLP-1 secretion through TGR5 signaling in diabetic patients. A combination of sitagliptin and UDCA is in phase-IV clinical trials [98]. The steroid agonist 6α-ethyl-23(S)-methylcholic acid (INT-777), specific to TGR5, has been found to promote thermogenesis and energy metabolism in adipose tissue [99]. Furthermore, it also stimulates the secretion of GLP-1 and inhibits macrophage-mediated inflammation and lipid accumulation [32], which can lead to improved insulin sensitivity and reduced hepatic steatosis [100]. Recent studies have shown that INT-777 inhibits neuroinflammation and improves nerve injury [101,102]. Meanwhile, in vivo studies using mouse model with NAFLD also validated the hypoglycemic effects of INT-777 [103].

RDX8940 is a highly effective and selective TGR5 agonist, which could promote the secretion of GLP-1, GLP-2, and PYY in mouse intestinal L cells with minimal systemic side effects. Unlike other agonists, it does not inhibit gallbladder emptying in mice, and it is also non-toxic to the heart, liver, and pancreas. Furthermore, RDX8940 improves hepatic steatosis and insulin sensitivity in insulin-resistant mouse models, which has the potential to treat NASH and NAFLD [14]. A-Ionone is an aromatic diterpenoid compound with the same TGR5-activating effect as CDCA and is used in food and cosmetics with a high safety coefficient. The administration of 0.2% α-Ionone to mice fed with HFD led to a significant rise in the expression of thermogenic genes, such as PGC-1α, UCP-1, and Dio2, in their brown adipose tissue. It inhibits weight gain in DIO mice by activating TGR5 compared to normal HFD mice. It is suggested that α-Ionone has shown potential as a TGR5 agonist for preventing obesity [104]. To improve TGR5 agonists for treating metabolic diseases, Zhao et al. [105] developed a new series of 1-benzyl–1H-imidazole–5-carboxyamide derivatives. The most potent compounds, 19d and 19e, activated TGR5 better than the control drugs INT-777 and LCA while exhibiting good FXR selectivity. In vivo studies also showed significant hypoglycemic effects.

Nevertheless, systemic TGR5 activation can cause some unpleasant adverse effects including excessive gallbladder filling and insufficient emptying [106,107]. TGR5 agonists have also been associated with “bile-acid-related” itching and analgesia [108]. Animal studies have illustrated that structurally diverse TGR5 agonists reduce vascular tone and blood pressure, resulting in reflex tachycardia and inotropy [109].

## 10. Conclusions

In summary, TGR5 activation influences glucolipid metabolism, energy metabolism, inflammatory response, and endogenous signaling molecules expression through multiple signal transduction pathways. It has excellent potential effects in treating T2DM, obesity, atherosclerosis, and NAFLD, which is a key direction and hot area for drug research and development at home and abroad. However, the activity of TGR5 has been demonstrated in animal and cellular assays and needs to be further validated and observed in clinical trials. A comprehensive understanding of the potential mechanisms of TGR5 in metabolic syndrome can serve as a theoretical foundation and reference value for future TGR5-specific targeting in the treatment of metabolic syndrome.

## Data Availability

Not applicable.
